# Evolutionary Relations of Hexanchiformes Deep-Sea Sharks Elucidated by Whole Mitochondrial Genome Sequences

**DOI:** 10.1155/2013/147064

**Published:** 2013-09-05

**Authors:** Keiko Tanaka, Takashi Shiina, Taketeru Tomita, Shingo Suzuki, Kazuyoshi Hosomichi, Kazumi Sano, Hiroyuki Doi, Azumi Kono, Tomoyoshi Komiyama, Hidetoshi Inoko, Jerzy K. Kulski, Sho Tanaka

**Affiliations:** ^1^Department of Molecular Life Science, Division of Basic Medical Science and Molecular Medicine, Tokai University School of Medicine, 143 Shimokasuya, Isehara, Kanagawa 259-1143, Japan; ^2^Fisheries Science Center, The Hokkaido University Museum, 3-1-1 Minato-cho, Hakodate, Hokkaido 041-8611, Japan; ^3^Division of Human Genetics, Department of Integrated Genetics, National Institute of Genetics, 1111 Yata, Mishima, Shizuoka 411-8540, Japan; ^4^Division of Science Interpreter Training, Komaba Organization for Education Excellence College of Arts and Sciences, The University of Tokyo, 3-8-1 Komaba, Meguro-ku, Tokyo 153-8902, Japan; ^5^Shimonoseki Marine Science Museum, 6-1 Arcaport, Shimonoseki, Yamaguchi 750-0036, Japan; ^6^Department of Clinical Pharmacology, Division of Basic Clinical Science and Public Health, Tokai University School of Medicine, 143 Shimokasuya, Isehara, Kanagawa 259-1143, Japan; ^7^Centre for Forensic Science, The University of Western Australia, Nedlands, WA 6008, Australia; ^8^Department of Marine Biology, School of Marine Science and Technology, Tokai University, 3-20-1 Orido, Shimizu, Shizuoka 424-8610, Japan

## Abstract

Hexanchiformes is regarded as a monophyletic taxon, but the morphological and genetic relationships between the five extant species within the order are still uncertain. In this study, we determined the whole mitochondrial DNA (mtDNA) sequences of seven sharks including representatives of the five Hexanchiformes, one squaliform, and one carcharhiniform and inferred the phylogenetic relationships among those species and 12 other Chondrichthyes (cartilaginous fishes) species for which the complete mitogenome is available. The monophyly of Hexanchiformes and its close relation with all other Squaliformes sharks were strongly supported by likelihood and Bayesian phylogenetic analysis of 13,749 aligned nucleotides of 13 protein coding genes and two rRNA genes that were derived from the whole mDNA sequences of the 19 species. The phylogeny suggested that Hexanchiformes is in the superorder Squalomorphi, *Chlamydoselachus anguineus* (frilled shark) is the sister species to all other Hexanchiformes, and the relations within Hexanchiformes are well resolved as *Chlamydoselachus*, (*Notorynchus*, (*Heptranchias*, (*Hexanchus griseus*, *H. nakamurai*))). Based on our phylogeny, we discussed evolutionary scenarios of the jaw suspension mechanism and gill slit numbers that are significant features in the sharks.

## 1. Introduction

The subdivision Selachii or modern sharks, along with skates and rays, comprises the subclass Neoselachii within the class Chondrichthyes or cartilaginous fishes. Chondrichthyans, including the Neoselachii, chimaeroids, and several fossil forms are defined as jawed fish with skeletons made of prismatic cartilage rather than bone and pelvic claspers in males. The Selachii can be divided into two superorders, the Galeomorphi (339 species) and the Squalomorphi (155 species), and eight extant orders [1].

Among the sharks, Hexanchiformes is regarded as an ancient order of sharks with just five extant species that are characterized by having only one dorsal fin, either six or seven gill clefts, and no nictitating membrane in the eyes [2]. The Hexanchiformes is usually divided into two families, the Chlamydoselachidae (*Chlamydoselachus anguineus *(*C. anguineus*)) and the Hexanchidae (*Hexanchus griseus* (*H. griseus*),* Hexanchus nakamurai* (*H. nakamurai*), *Notorynchus cepedianus *(*N. cepedianus*), and *Heptranchias perlo *(*H. perlo*)) with the latter family also known as “cow sharks.” The frilled shark, *C. anguineus*, is very different from the cow sharks, and its own order of Chlamydoselachiformes was proposed [3]. However, derived features (e.g., the extra gill arch and more-heart valve rows) shared with other Hexanchiformes support its retention within the Hexanchiformes. A third family, Notorynchidae, was also proposed for the *Notorynchus *species because of morphological and behavioral differences from the other members of the family Hexanchidae [3]. Interestingly, the tooth structure and composition of one of the Hexanchiformes, *C. anguineus*, is similar to that of the stem-group fossil shark *Cladoselache* sp., although such features are not observed in the other Hexanchiformes species [4]. Therefore, the accurate placement of Hexanchiformes is essential to understand the evolution of morphology in sharks. However, the lack of available DNA sequence data for most shark species and orders remains a major limitation to obtaining reliable results in molecular phylogenetic studies. 

The mitochondrial DNA (mtDNA) has been one of the most widely used molecular markers for diversity and phylogenetic studies in animals because of its size, maternal mode of inheritance, high rate of mutation, and simple genomic structure [5]. Although mtDNA sequences have proved valuable in determining phylogenetic relationships, the choice of a gene as a molecular marker and clock in phylogeny is also important [6, 7]. Recent phylogenetic studies in different taxa suggest that full-length mitochondrial genomic sequences provide an improved resolution for reconstructing a robust phylogeny and for molecular dating of divergence events within a phylogeny [8]. So far, of all the shark species, the complete mtDNA sequences were determined in the subclass Neoselachii of some species, and the sequences were used for elucidating interrelationships between sharks and bony fishes and between sharks and rays [9–14]. Therefore, there were no analyses of the relationships between species within cartilaginous fish orders using whole mtDNA sequences, although intrarelationships were estimated by using partial mtDNA and nuclear DNA sequences [6, 7, 10, 15–20].

In order to obtain more sequence data for different shark species and to allow accurate placement within the order Hexanchiformes, we chose to determine the complete mtDNA sequences of seven shark species including five species of the order Hexanchiformes, along with *Somniosus pacificus* (*S. pacificus*), which is a member of the order Squaliformes and *Pseudotriakis microdon* (*P. microdon*), which is a member of the order Carcharhiniformes. We then analysed the phylogenetic relationships among the five Hexanchiformes species and between the Hexanchiformes and ten other shark species using the complete mitochondrial genomic sequences of 19 cartilaginous fish species. On the basis of the results of our phylogenetic analysis, we propose new scenarios for the evolution of the jaw suspension mechanism and the number of gill clefts that are significant features in sharks.

## 2. Materials and Methods

### 2.1. Sample Collection and Isolation of the mtDNA

**Figure 1:**
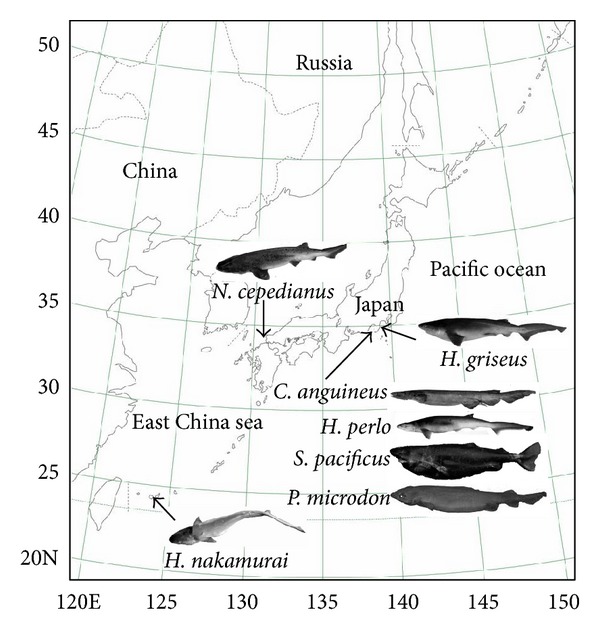
Geographic locations of the five Hexanchiformes species and two other species caught off the coast of Japan for nucleotide sequencing in this study. Biological features of the sharks are shown in Table 1.

The shark specimens in this study were all captured off the coast of Japan, *C. anguineus*, *H. perlo, S. pacificus*, and *P. microdon* within Suruga Bay, *H. griseus* within Sagami Bay, *H. nakamurai *near Ishigaki Island, and *N. cepedianus* near Futaoi Island (Figure 1). In this paper, we use the species name *Hexanchus nakamurai *[21] instead of its synonym* Hexanchus vitulus *[22]. The mtDNAs were isolated from the muscle or spleen tissue using the mtDNA Extractor CT Kit (Wako Pure Chemical Industries, Ltd., Osaka, Japan) or standard phenol-chloroform method [23]. The quantity and quality of the isolated DNA samples were measured and estimated, respectively, by the spectral absorbance of the DNA at 260 nm and 280 nm.

### 2.2. PCR Amplification of the Entire mtDNA Regions

Twenty-two pairs of primers were newly designed by comparing previously published shark mtDNA sequences in the GenBank/EMBL/DDBJ database with assistance from Primer Express v1.0 (Applied Biosystems, CA, USA) for polymerase chain reaction (PCR) amplification of the entire mtDNA regions (eee Supplementary Table 1 in Supplementary Material available online at http://dx.doi.org/10.1155/2013/147064). For PCR amplification of the cytochrome b (CYTB) region, the 10 *μ*L amplification reaction contained 10 ng of mtDNA, 1.0 unit of Ex Taq polymerase (TaKaRa Shuzo, Otsu, Japan), 1x PCR buffer, 2.5 mM MgCl_2_, 2 mM of each dNTP, and 0.5 *μ*M of each primer. The cycling parameters were as follows: an initial denaturation of 96°C for 3 min followed by 30 cycles of 96°C for 30 sec, 50°C for 30 sec, and 72°C for 1 min and followed by a final cycle of 72°C for 4 min. For long-ranged PCR amplifications, the 20 *μ*L amplification reaction contained 10 ng of mtDNA, 0.4 unit of KOD-FX polymerase (TOYOBO, Osaka, Japan), 2x PCR buffer, 2.5 mM MgCl_2_, 400 *μ*M of each dNTP, and 0.5 *μ*M of each primer. The cycling parameters were as follows: an initial denaturation of 94°C for 2 min followed by 35 cycles of 98°C for 10 sec, 60 or 68°C for 30 sec, and 68°C for 2 to 20 min. PCR reactions were performed by using the thermal cycler GeneAmp PCR system 9700 (Applied Biosystems, CA, USA). The long-ranged PCR size was 6.5 kb on average and ranged from 1,711 bp to 16,800 bp (Supplementary table 1).

### 2.3. Genomic Sequencing Strategy and Sequence Analysis

The PCR products were subjected to complete and bidirectional shotgun sequencing with an average 7.2x coverage which was sufficient for assembly and analysis of the entire sequence using previously established procedures [26] and direct sequencing using PCR primers as sequencing primers. DNA sequencing was performed by the cycle sequencing method using Ampli*Taq*-DNA polymerase FS and the fluorescently labeled BigDye terminators in a GeneAmp PCR system 9700 (Applied Biosystems, Foster City, CA, USA). A 3130xl Genetic Analyzer was used for automated fluorescent sequencing (Applied Biosystems, Foster City, CA, USA). Individual sequences were minimally edited to remove vector sequences and assembled into contigs using the Sequencher 4.2 software (Gene Codes CO., MI, USA). Remaining gaps or ambiguous nucleotides were determined by the direct sequencing of PCR products obtained with appropriate PCR primers or by nucleotide sequence determination of shotgun clones.

Nucleotide similarities between sequences were calculated by the “Search Homology” tool of GENETYX-MAC ver. 12.0 (Software Development Co. Ltd., Tokyo, Japan), and those with nucleotide sequences in GenBank/EMBL/DDBJ were searched by BLAST program (http://www.ncbi.nlm.nih.gov/BLAST/). The newly determined mtDNA sequences were annotated by comparison with known mtDNA sequence information of other shark species.

### 2.4. Phylogenetic Analysis

Multiple sequence alignments were created using the ClustalW Sequence Alignment program of the Molecular Evolution Genetics Analysis software 5 (MEGA5; [27]). Nucleotide alignments were separately created for each of the 13 protein coding genes, *ND1, ND2, COX1, COX2, ATP8, ATP6, COX3, ND3, ND4L, ND4, ND5, ND6*, and *CYTB* and two ribosomal RNA (rRNA) genes, *12S* and *16S*. After excluding some gaps, 13,749 nucleotides were aligned (Align_Set_1) for 19 Chondrichthyes species (including *C. monstrosa*), and 13,784 nucleotides were aligned (Align_Set_2) for 18 Selachii and Batoidea species using the ClustalW sequence alignment settings with few manual adjustments (Table 2).

Phylogenetic trees were constructed using the model-based maximum likelihood (ML) and Bayesian inference (BI: MrBayes Ver. 3.2.1 [28]) methods and the distance-based neighbour-joining (NJ) method (MEGA5). For the ML analyses we used “Find best DNA/protein models (ML)” program of MEGA5 to estimate the most likely model of sequence evolution including third codon sites. Based on maximum likelihood values and the Akaike information criterion (AIC) [29], the TN93+G+I model was selected as the most likely model (ln⁡*L* = −118970.445, AIC = 238464.763 for Align_Set_1, and ln⁡*L* = −110086.677, AIC = 220253.367 for Align_Set_2) for a nucleotide based ML tree using 10,000 ML-bootstrap replicates. The neighbor-joining tree was constructed using distances corrected according to the Kimura 2-parameter model with 1.0 gamma parameters [30] and assessed using 10,000 bootstrap replicates. For the BI analyses, we used MrAIC Ver. 1.4.4, http://www.abc.se/~nylander/, Nylander unpublished) with PhyML Ver. 3.0 [31] to estimate the most likely model of the sequence evolution. Based on maximum likelihood values and the Bayesian information criterion (BIC), the GTR+G+I model was selected as the most likely model (ln⁡*L* = −118104.860, BIC = 236638.513 for Align_Set_1 and ln⁡*L* = −109301.198, BIC = 218688.396 for Align_Set_2) for the Bayesian inference (BI) method. The Bayesian analysis was run using the Metropolis coupled Markov chain Monte Carlo (MCMC) algorithm from randomly generated starting trees for 1,500,000 generations with sampling every 100 generations. The first 100,100 steps of each run were discarded as burn-in. The stabilized burn-in level was assessed using Tracer v1.4 (http://beast.bio.ed.ac.uk/Tracer; Rambaut & Drummond unpublished). Convergence for both runs was examined using the average standard deviation of the split frequencies and through examination of the Markov Chain Monte Carlo chains using Tracer v1.4.

### 2.5. Estimation of Divergence Times

Divergence times for each node of Hexanchiformes and other sharks from the orders Squalomorphi and Galeomorphi were estimated by the Divergence Time program in MEGA5 software based on the phylogenetic tree of the ML method and on previously estimated divergence times of Selachii and Batoidea (213.4 Mya (203.3~228.8 Mya in the 95% confidence intervals)) [24].

## 3. Results

### 3.1. Biological and Genetic Information

The biological and genetic features of the seven shark species sampled for this study are shown in Table 1. Six or seven gill clefts and one dorsal fin were observed in the Hexanchiformes species, whereas five gill clefts and two dorsal fins were observed in *S. pacificus *and *P. microdon* and in all non-Hexanchiformes and Neoselachii, except some skates and myliobatoid rays in which dorsal fins were presumably lost secondarily [32]. Our preliminary sequence analysis of the mtDNA *Cytb* or *12S* genes for six of the seven species, excluding *H. nakamurai*, using a BLAST search (GenBank) primarily showed the highest nucleotide identities (97.0% to 100%) with previously published nucleotide sequences of the target species and secondarily showed lower nucleotide identities (80.2% to 93.1%) with the nucleotide sequences of different species (Table 1). Therefore, this analysis, on the basis of biological and/or genetic features, helped us to cross-check and confirm that all our collected samples were identified correctly as the targeted species.

### 3.2. Genome Structure of mtDNA Sequences in Chondrichthyes Species

Whole mtDNA sequences of the seven sharks were determined by PCR-based shotgun sequencing. Their nucleotide length was 17,314 bp in *C. anguineus*, 16,990 bp in *N. cepedianus*, 18,909 bp in *H. perlo*, 17,223 bp in *H. griseus*, 18,605 bp in *H. nakamurai*, 16,730 bp in *S. pacificus*, and 16,700 bp in *P. microdon *(GenBank/EMBL/DDBJ accession numbers: AB560487 to AB560493), with the mtDNA length variability due to the presence of varying numbers and compositions of tandem repeats in the control region (Supplementary table 2). From a genomic comparison of 19 Chondrichthyes mitogenomes, all were basically composed of two rRNAs (*12S* and *16S*), 22 transfer RNAs (tRNAs), 13 protein coding genes, and the D-loop control region. Species-specific duplications, insertions, and deletions were observed in some species such as the duplication of tRNA-Trp and the deletion of tRNA-Asn and tRNA-Leu2 in *M. manazo* and insertion of tRNA-Thr for the D-loop region in *C. monstrosa *(Supplementary table 2). The gene directions of *ND6* and eight tRNAs, tRNA-Gln, tRNA-Ala, tRNA-Asn, tRNA-Cys, tRNA-Tyr, tRNA-Ser, tRNA-Glu, and tRNA-Pro, were encoded in the mtDNA L chain, and the other genes were encoded in the H chain in all species. The GC contents of the species ranged from 35.0% in *C. anguineus* to 42.4% in *O. kenojei *(Supplementary table 2).

### 3.3. Phylogeny of the Hexanchiformes Using Complete mtDNA Sequences

**Figure 2:**
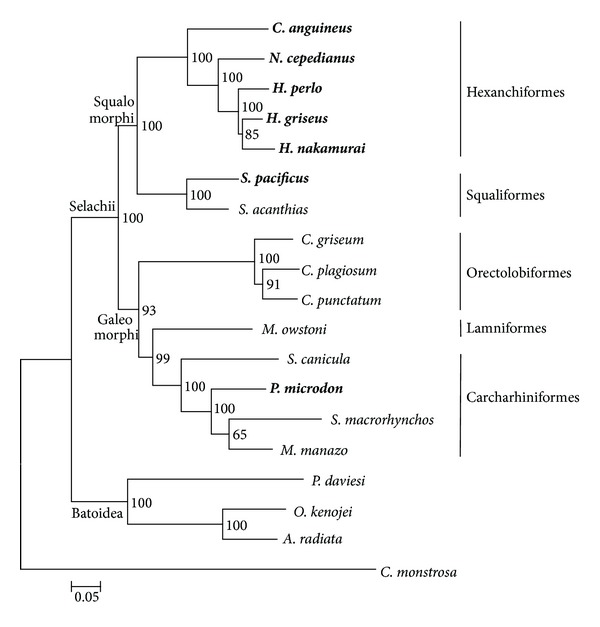
Maximum likelihood phylogeny depicting relationships among 19 Chondrichthyes species inferred from the whole-mtDNA sequences. The 13,749 bp nucleotide alignment (Align_Set_1) was used for the analysis. Numbers on the branches are bootstrap support values. Bold letters indicate the species that were newly sequenced for this study. The Neoselachii subdivisions (Selachii, Batoidea), superorders (Squalomorphi, Galeomorphi) and orders (Hexanchiformes, Squaliformes, Orectolobiformes, Carcharhiniformes, and Lamniformes) are indicated in block letters on the basal branches of the tree. The outgroup species *C. monstrosa* is in the subclass Holocephali.

Figure 2 shows an ML phylogenetic tree constructed by using the TN93+G+I model of the ML method. The tree was constructed using the 13,749 bp nucleotide alignment (Align_Set_1) of the 13 protein coding genes and two rRNA genes from 19 species with *C. monstrosa *selected as the outgroup. In the aligned sequence 48.7% nucleotides (6,693 sites) were constant sites. The similar topology, supported clades, and bootstrap support values or posterior probability were shown by the GTR+G+I model of the BI method and the Kimura 2-parameter model of the NJ method using the same nucleotide alignment as for the ML method (Supplementary figure 1). Moreover, even if we set three Batoidea species as outgroups, the same topology, supported clades, and bootstrap support values or posterior probability were shown by the TN93+G+I model within the ML method, the GTR+G+I model within the BI method and the Kimura 2-parameter model within the NJ method using 13,784 nucleotide alignment (Align_Set_2) of the 13 protein coding genes and two rRNA genes from 18 species (Supplementary figure 2). 

Our phylogenetic analyses of nucleotide sequences using the ML, BI, and NJ methods suggest that the Selachii is divided into the two superorders, Squalomorphi and Galeomorphi. The Squalomorphi superorder includes the orders Hexanchiformes and Squaliformes, and the Galeomorphi superorder includes the orders Orectolobiformes, Lamniformes, and Carcharhiniformes (Figure 2). In Figure 2 tree, the Hexanchiformes clade is monophyletic and separates from the Squaliformes clade in the Squalomorphi order that is separated from the Galeomorphi lineage. The Hexanchiformes and Squaliformes lineages showed clades with extremely high bootstrap support values (100) and posterior probabilities (100) (Figure 2, Supplementary figures  1 and  2). The mitogenomic data also show good resolution within the Galeomorphi with Orectolobiformes separated from “Lamniformes plus Carcharhiniformes” with high bootstrap support values (61~93) and posterior probabilities (100) (Figure 2, Supplementary figures  1 and  2).

Within the Hexanchiformes, *C. anguineus* is sister of all the others with the longest branch, so, assuming clock like evolution, it could be the oldest extant shark lineage. The *N. cepedianus* lineage emerged next. By comparison, the terminal branch lengths of *H. griseus*, *H. nakamurai*, and *H. perlo* are relatively short.

**Figure 3:**
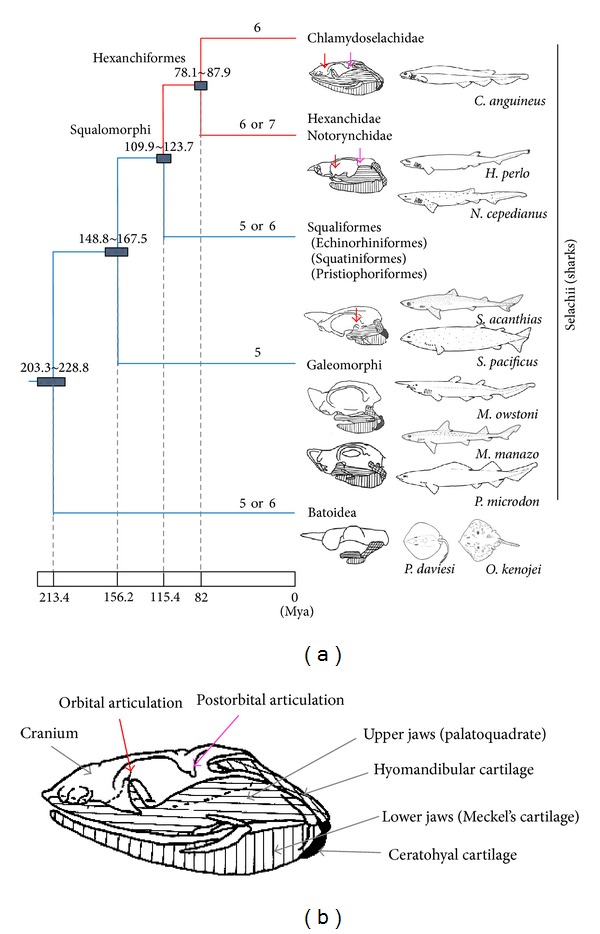
Morphological character evolution mapped onto the phylogenetic tree derived in this study (a) and structure of jaw suspension of *C. anguineus* (b). (a) Divergence time for each node of Selachii was estimated by the divergence time of Selachii and Batoidea (213.4 Mya (203.3~228.8 Mya in the 95% confidence intervals)) [24]. The red line extending from the circled node indicates the evolutionary time (115.4 Mya) of the divergence of Hexanchiformes from the other Squalomorphi lineages. Numbers above the branches indicate the gill cleft numbers, and numbers above the nodes indicate range of the divergence time. The schematic diagrams of jaw suspension were based on previous reports [3, 13, 25]. (b) Vertical, horizontal, and grid-lined areas and black and white areas indicate lower jaws (Meckel's cartilage), upper jaws (palatoquadrate), hyomandibular and ceratohyal cartilages, and cranium, respectively. Red and orange arrows in the schematic diagram of the jaw suspension apparatus indicate orbital and postorbital articulations, respectively.

Assuming the divergence time of Selachii and Batoidea was 213.4 Mya (203.3 Mya~228.8 Mya) [24] and based on divergence rates in Figure 2, then the divergence times for each node of Squalomorphi and Galeomorphi and Squaliformes and Hexanchiformes are estimated as 156.2 Mya (148.8 Mya~167.5 Mya) and 115.4 Mya (109.9 Mya~123.7 Mya), respectively (Figure 3(a)). Of the Hexanchiformes, the divergence time of* C. anguineus* is estimated as 82.0 Mya (78.1 Mya~87.9 Mya). This estimation is largely consistent with the known fossil record of Chlamydoselachiformes (85 Mya) [33, 34]. 

## 4. Discussion

### 4.1. Phylogeny of Cartilaginous Fishes

In the case of Batoidea, Carcharhiniformes, Hexanchiformes, and Squaliformes, our phylogeny supports, in a number of respects, the previous findings derived from partial mitochondrial genomes, genes *COX1, NADH2, NADH4, CYTB, 12S, 16S*, and/or tRNAs and nuclear genome genes *5.8S, 18S, 28S*, and *RAG1* [6, 7, 15–19, 35]. Recently, Naylor et al. [7] published the most comprehensive phylogeny of sharks using a total of 595 shark species representing eight orders and 159 genera and 56 families, but mainly using a single mitochondrial gene (*NADH2*) as the molecular marker. Although we did not have any examples of the Echinorhiniformes, Pristiophoriformes, and Squatiniformes in our study, the remaining orders within Squalomorphi were generally similar to the relationships reported by Vélez-Zuazo [6] and by Naylor et al. [7] of Hexanchiformes and Squaliformes within Squalomorphi and Lamniformes, Orectolobiformes, and Carcharhiniformes within Galeomorphi. Recent myological studies on Hexanchiformes also support the inclusion of the Chlamydoselachidae and Hexanchidae in the Squalomorphi [36].

Our analysis, though limited to just one lamniform representative, found strong support for Lamniformes as the sister order of Carcharhiniformes [2, 19] instead of the Orectolobiformes [6, 35]. In addition, our phylogeny is inconsistent with some previously reported morphology-based phylogenies such as the hypnosqualean hypothesis that places the batoids within sharks [2, 37], the tooth structure-based tree that places Hexanchiformes outside of batoids [38, 39], the jaw protrusion, and feeding-based trees [40] and a Bayesian analysis based on the CYTB gene that supported the position of Hexanchiformes as the sister of all other shark orders [20]. The full-length mitochondrial genomic sequences provide strong statistical support and an improved resolution for reconstructing a robust phylogeny in the cartilaginous fish [8]. Therefore, our phylogeny appears to be a reasonable reconstruction of the evolutionary process dividing the Selachii into the two superorders, Squalomorphi and Galeomorphi. However, the divergence time for a node of Squaliformes and Hexanchiformes was estimated to be 115.4 Mya (109.9 Mya~123.7 Mya). This is not largely consistent with the known fossil record of Hexanchiformes (190 Mya) [33]. In this regard, in case of setting the divergence time of Hexanchiformes for 190 Mya, the divergence times for each node of Selachii/Batoidea and Chlamydoselachidae were estimated as 134.9 Mya and 351.3 Mya, respectively. The divergence time of Chlamydoselachidae is consistent with the known fossil record of Chlamydoselachidae (85 Mya) [33] but that of Selachii/Batoidea is not consistent with the previously estimated divergence time of Selachii and Batoidea (213.4 Mya (203.3~228.8 Mya in the 95% confidence intervals)) [24]. In this study we used only 15 Selachii and three Batoidea species for the phylogenetic analysis, but some Selachii species that show ambiguous classification such as Echinorhiniformes, Pristiophoriformes, and Squatiniformes were not included in this study. Therefore, detailed phylogenetic analysis based on full-length mitochondrial genomic sequences using additional Selachii species that are thought to be diverged on evolutionary important positions, will be necessary for estimation of the precise divergence times in future.

### 4.2. Phylogenetic Relationships in Hexanchiformes

Vélez-Zuazo and Agnarsson [6] reported that the Hexanchidae within Hexanchiformes was paraphyletic because it also contained the only species of Chlamydoselachidae. Their Bayesian analysis of Hexanchiformes, in using only a small portion (15%) of a single nucleotide sequence composed of five genes, *COX1, NADH2, CYTB, 16S*, and *Rag1*, may be compromised by too little data. They showed low support with a posterior probability of 45% at the node splitting the *C. anguineus* and *N. maculates* taxa. In addition, although they found that *N. maculates* was a sister group to the *N. cepedianus*, *N. maculates* is in fact a synonym of *N. cepedianus* and therefore the same species [6]. In our analysis, *N. cepedianus* is clearly the sister of the *Heptranchias-Hexanchus* clade with which it forms a clade separate from *Chlamydoselachus* with all nodes strongly supported (Figure 2). The sequencing of the complete mitochondrial genome of *N. maculates,* if it is a separate species from *N. cepedianus, *should help to resolve this issue.

The frilled shark, *C. anguineus*, in our phylogenetic analysis, was found to be the sister group to all the other Hexanchiformes, similar to the results on their anatomical studies [2, 3, 36]. Some systematists have proposed a separate order for the frilled shark [3]. Recently, a new Chlamydoselachidae species (*Chlamydoselachus africana; C. africana*) was discovered around southern Africa [41]. Although it is not known if the mtDNA of *C. africana* will group phylogenetically with *C. anguineus,* our phylogeny supports the retention of *C. anguineus* within the Hexanchiformes rather than within its own order. The frilled shark, *C. anguineus,* is placed in its own family, Chlamydoselachidae, because of its many unusual features such as elongated and eel-like body; its low dorsal fin; blunt snout, long jaws that are narrower at the tip than at the corners; terminal mouth, similar upper and lower teeth with three prong-like cusps; and gill clefts with frilly margins and the first gill slit continuous across the throat [42]. 

The position of *N. cepedianus* in our phylogenetic tree favours its own family name of Notorynchidae rather than being placed into the Hexanchidae family with the one *Heptranchias* species and the two *Hexanchus* species. Incidentally, *H. nakamurai* [21] in our study corresponds to *H. viulus* [22] in the Vélez-Zuazo and Agnarsson study [6], as the latter species name is junior to the former name [42, 43]. However, Naylor et al. [44] suggest that both *H. nakamurai* and *H. viulus* may actually represent valid species.

### 4.3. New Insights on the Morphological Evolution of Sharks

Our phylogenetic analysis based on mitochondrial genomic sequences is useful for comparing morphological features to phylogenetic relationships among the sharks. For example, the Hexanchiformes species have six or seven gill clefts and one dorsal fin, whereas most of other Selachii species have five or six gill clefts and mostly two dorsal fins (Table 1). Most fossils of Agnathans and some fossils of acanthodian (stem Chondrichthyes or stem osteichthyes) support the presence of multiple gill clefts [45], and the sea lamprey, which is in the class Petromyzontida, has seven gill clefts. However, the rabbitfish, which is in the subclass Holocephali, is from the sister clade of sharks and Batoidea, and it has one gill cleft. In this regard, the phylogenetic tree suggests that the multiple gill clefts have been maintained by species-specific increases and decreases in both the Petromyzontida and Neoselachii lineages (Figure 3(a)), but holocephalians could easily have reduced gill slits to one.

The phylogenetic placement of Hexanchiformes in our study suggests a clearer scenario for the evolution of the upper jaw suspension in sharks. Upper jaws (palatoquadrate) of sharks are “suspended” from the cranium by the hyomandibular cartilage and several articulations between the cranium and upper jaw (Figure 3(b)) and shark jaws evolved with a general trend towards jaw shortening, increased kinesis of upper jaw suspension, and protrusion [46]. Many researchers have focused on the evolution of the jaw suspension mechanism in order to clarify the phylogenetic relationship within sharks [47–49]. Because the jaw suspension mechanism is strongly coupled with the jaw protrusion capability, the evolution of jaw protrusion behaviour has been reconstructed based on the evolution of jaw suspension mechanisms [46]. However, the position of Hexanchiformes as the sister to Squaliformes (Figure 3) suggests that the Hexanchiformes represents an evolved state of the jaw suspension mechanism independent of the other shark orders. It can be expected that investigation of fossils from non-Neoselachii Chondrichthyes such as the hybodonts and cladodonts will help to elucidate the polarization of the evolution of the jaw suspension mechanism.

The Hexanchiformes species have two characteristic articulations between their cranium and palatoquadrate. One is the orbital articulation, which is between the orbital process of the palatoquadrate and the orbital walls of the cranium, and the other is the postorbital articulation, which is between the otic process of the palatoquadrate and the postorbital process of the cranium [47, 48]. Due to the postorbital articulation, jaw protrusion capabilities of the Hexanchiformes species are strongly restricted. On the other hand, Squaliformes species lack the postorbital articulations, and Galeomorphi species lack both the orbital articulations and the postorbital articulation [47, 48]. Because the postorbital and/or orbital articulations are absent from these two clades, in contrast to Hexanchiformes species, they have a greater capability of jaw protrusion, flexibility, and maneuverability [46]. Extensive upper jaw protrusion in modern sharks was found to allow faster closure of jaws to gouge or cut smaller pieces of prey to fit into the mouth [50]. Therefore, based on the differences in jaw suspension mechanisms among Hexanchiformes, Squaliformes, and Galeomorphi, the following evolutionary scenario can be proposed. First, the orbital process and postorbital articulation were missing from the common ancestor of all shark orders. Second, the orbital articulation was gained at the base of Squaliformes + Hexanchiformes clade, and this modification restricted the evolved sharks to protrude their jaws. Third, the postorbital articulation was gained at the base of Hexanchiformes clade, and this modification might have further restricted the Hexanchiformes from the capability of upper jaw protrusion.

Previous research suggested that the presence of the orbital process in the palatoquadrate is one of the strong morphological characteristics indicating the monophyly of Hexanchiformes and Squaliformes. Thus, Maisey named the Hexanchiformes + Squaliformes clade as “orbitostylic sharks” [25, 47–49]. Our result suggests that the presence of the orbital process is an evolved character of the Hexanchiformes + Squaliformes clades and not a plesiomorphic character of the whole shark clade (Figure 3(a)). 

In summary, we sequenced and analysed the complete mtDNA sequences of five Hexanchiformes species. Our phylogeny and the known morphological features of sharks resolved interrelationships of major Hexanchiformes species. Further insights into phylogeny of the mtDNA sequences were provided by comparative analyses using other shark and nonshark species. A similar approach using the whole mtDNA genome of sharks in the other orders should help to resolve the intraspecific and interspecific relationships within Chondrichthyes (cartilaginous fishes). The Hexanchiformes are observed mainly in deep-sea areas (<1000 m) all over the world. However, they rarely occur in the continental shelf shallower than 200 m [51]. This may be a relic of a past behavioral habit, when their ancestors had inhabited the coastal shelf [52, 53]. Although some Hexanchiformes specific morphological features such as tooth, gill cleft numbers, jaw suspension, and no nictitating membrane in the eyes. have been reported so far, the other features such as distribution of the living areas, physiology, reproduction and genetic diversity are unknown. Therefore, it will be necessary to compare between those features and phylogenetic relationships derived from nucleotide sequences to comprehensively understand evolution of the sharks including Hexanchiformes. 

## Supplementary Material

The supplementary material contains Phylogenetic trees depicting genetic relationships using Align_Set_1 and Align_Set_2, Primer information used for the PCR amplification of mitochondrial genomes, and Structural features of mitochondrial genomes in Chondrichthyes.

## Figures and Tables

**Table 1:** Biological features of seven sharks analyzed in this study.

Scientific name		*C. anguineus *	*N. cepedianus *	*H. perlo *	*H. griseus *	*H. nakamurai *	*S. pacificus *	*P. microdon *
Captured location		Suruga Bay	Futaoi Island	Suruga Bay	Sagami Bay	Ishigaki Island	Suruga Bay	Suruga Bay
Captured date		25 Sep. 2007	5 Feb. 2008	26 Sep. 1996	9 Jul. 2008	5 Sep. 2009	31 Mar. 2009	13 Oct. 2008
Sex		F	M	F	F	M	M	F
Total length (mm)		1,480	1,500	715	4,270	1,300	2,900	2,084
Body length (mm)		1,226	Un	483	Un	Un	2,450	1,685
Body weight (g)		10,400	17,000	1,272	Un	7,000	Un	43,040
Gill cleft number		6	7	7	6	6	5	5
Dorsal fin number		1	1	1	1	1	2	2
Nucleotide identity with previously published mtDNA nucleotide sequences	Primary	99.7% with *C. anguineus* CYTB (D50022)	98.7% with *N. cepedianus* CYTB (M91186)	97.3% with *H. perlo* 12S rRNA (AY14788)	100% with *H. griseus* CYTB (DQ132493)	*H. nakamurai * mtDNA sequences have not been published so far.	100% identity with *S. pacificus* CYTB (EF090957)	97.0% with *P. microdon* CYTB (DQ422078)
	Secondary	80.2% with *M. pelagios* CYTB (U91440)	87.8% with *H. griseus* CYTB (DQ132493)	93.1% with *H. griseus* 12S rRNA (AY147887)	82.0% with *C. anguineus* CYTB (D50022)	90.9% with *H. griseus* CYTB (DQ132493)	84.2% with *S. acanthia* CYTB (Y18134)	89.9% with *G. attenuatus* CYTB (DQ422079)

Un: the feature is not known.

**Table 2:** List of the 19 mitochondrial genomes analyzed in this study.

Classification	Scientific name	Common name	Accession no. and reference
Chondrichthyes			
Neoselachii			
Selachii			
Squalomorphi			
Hexanchiformes			

** Chlamydoselachidae **	***Chlamydoselachus anguineus***	**Frilled shark**	**AB560487, this study**
** Hexanchidae**	***Heptranchias perlo***	**Sharpnose sevengill shark**	**AB560489, this study**
	***Hexanchus griseus***	**Bluntnose sixgill shark**	**AB560490, this study**
	***Hexanchus nakamurai***	**Bigeye sixgill shark**	**AB560491, this study**
** Notorynchidae**	***Notorynchus cepedianus***	**Broadnose sevengill shark**	**AB560488, this study**

Squaliformes			
Squalidae	*Squalus acanthias *	Spiny dogfish	Y18134, Rasmussen and Arnason [11]

** Somniosidae**	***Somniosus pacificus***	**Pacific sleeper shark**	**AB560492, this study**

Galeomorphi			
Orectolobiformes			
Hemiscylliidae	*Chiloscyllium plagiosum *	Whitespotted bamboo shark	FJ853422, [54] Zhang et al.
	*Chiloscyllium griseum *	Grey bamboo shark	JQ434458, unpublished
	*Chiloscyllium punctatum *	Brownbanded bamboo shark	JQ082337, Chen et al. [55]
Carcharhiniformes			
Carcharhinidae	*Scoliodon macrorhynchos *		JQ693102, Chen et al. [56]
Triakidae	*Mustelus manazo *	Starspotted smooth-hound	AB015962, Cao et al. [9]
Scyliorhinidae	*Scyliorhinus canicula *	Small spotted catshark	Y16067, Delarbre et al. [10]

** Pseudotriakidae**	***Pseudotriakis microdon***	**False catshark**	**AB560493, this study**

Lamniformes			
Mitsukurinidae	*Mitsukurina owstoni *	Goblin shark	EU528659, unpublished
Batoidea			
Rajiformes			
Rajidae	*Okamejei kenojei *	Ocellate spot skate	AY525783, Kim et al. [14]
	*Amblyraja radiata *	Starry ray	AF106038, Rasmussen and Arnason [12]
Myliobatiformes			
Plesiobatidae	*Plesiobatis daviesi *	Deepwater stingray	AY597334, unpublished
Holocephali			
Chimaeriformes			
Chimaeridae	*Chimaera monstrosa *	Rabbitfish	AJ310140, Arnason et al. [13]

Note-Classifications follow Nelson (2006) and Inoue et al. [8]. Bold letter indicates the mitochondrial genome sequences of shark species determined in this study.
