# Supplementary material for: Evolutionary Relations of Hexanchiformes Deep-Sea Sharks Elucidated by Whole Mitochondrial Genome Sequences
Source: Biomed Res Int. 2013 Sep 5;2013:147064. doi: 10.1155/2013/147064 (PMC3780621; doi:10.1155/2013/147064)
Supplement: Supplementary file 1 — The supplementary material contains Phylogenetic trees depicting genetic relationships using Align_Set_1 and Align_Set_2, Primer information used for the PCR amplification of mitochondrial genomes, and Structural features of mitochondrial genomes in Chondrichthyes. [file 147064.f1.pdf]

Supplementary figure 1

Neighbour-joining (A) and Bayesian inference (B) phylogenetic trees depicting genetic relationships among 19 Chondrichthyes species inferred from the whole mtDNA region. The 13,749 bp nucleotide alignment (Align_Set_1) was used for the analysis. Numbers showing on the branches are bootstrap support values in A and posterior probability in B. Bold letters indicate the nucleotide sequences of the species that we newly determined in this study.

Supplementary figure 2

Maximum likelihood (A), Neighbour-joining (B) and Bayesian inference (C) phylogenies depicting genetic relationships among 18 Selachii and Batoidea species inferred from the whole mtDNA region. The 13,784 bp nucleotide alignment (Align_Set_2) was used for the analysis. Numbers showing on the branches are bootstrap support values in A and B and posterior probability in C. Bold letters indicate the nucleotide sequences of the species that we newly determined in this study.

Supplementary figure 1

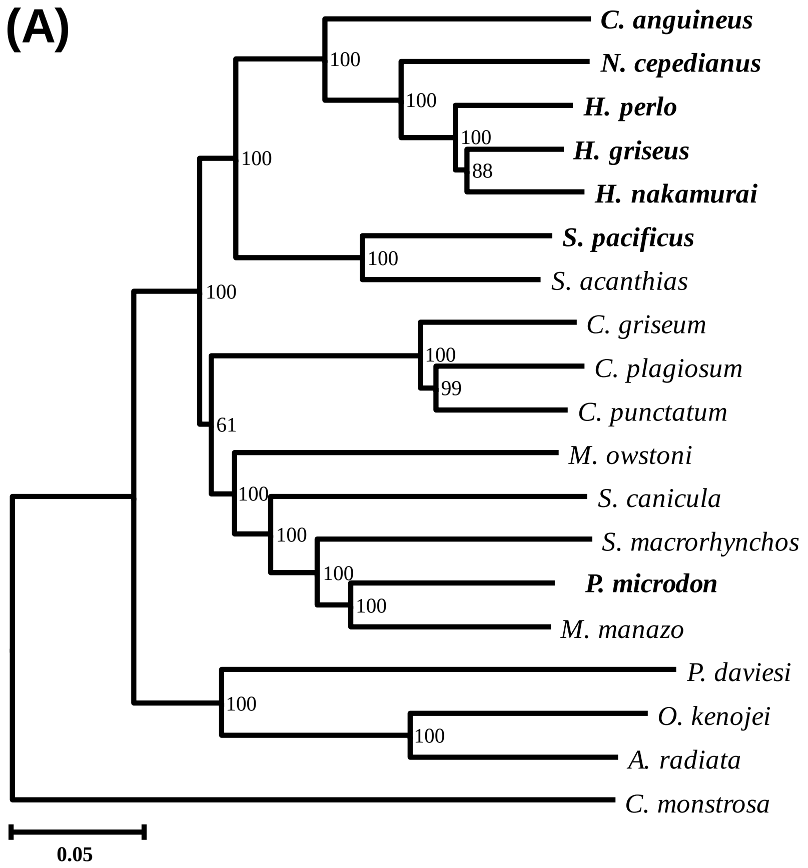

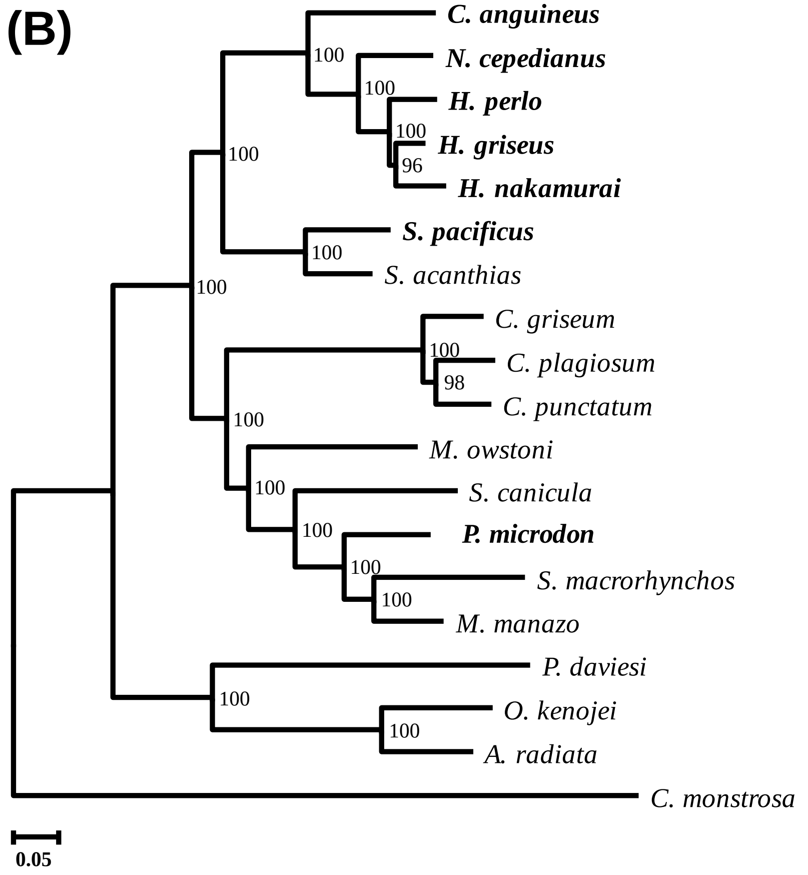

Supplementary figure 2

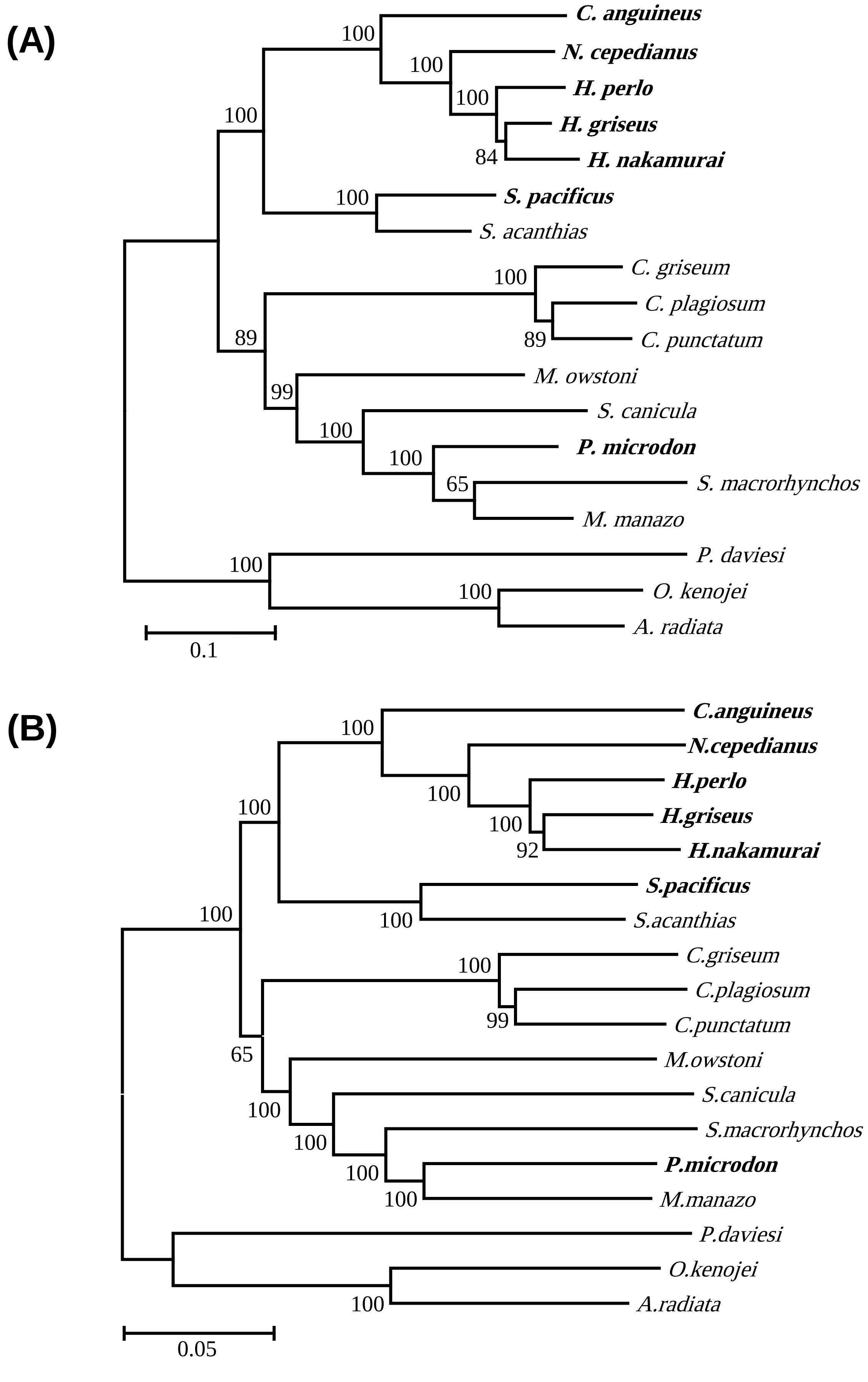

(C)

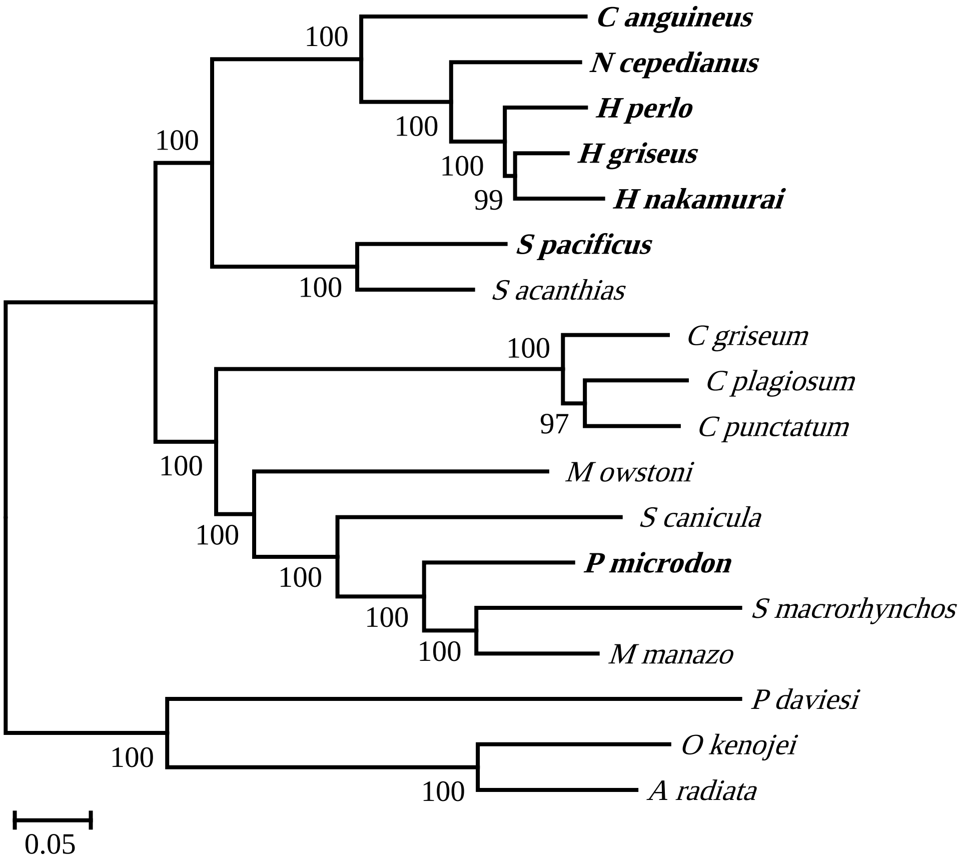
C anguineus
N cepedianus
H perlo
H griseus
H nakamurai
S pacificus
S acanthias
C griseum
C plagiosum
C punctatum
M owstoni
S canicula
P microdon
S macrorhynchos
M manazo
P daviesi
O kenojei
A radiata
100
100
100
100
99
100
100
97
100
100
100
100
100
100
100
0.05
